# Multiple Cutaneous Nodules in an HIV-Infected Patient

**DOI:** 10.1371/journal.pntd.0003291

**Published:** 2014-12-11

**Authors:** Atchara Phumee, Sarunyou Chusri, Kanyarat Kraivichian, Jade Wititsuwannakul, Thanaporn Hortiwakul, Usavadee Thavara, Khachornsakdi Silpapojakul, Padet Siriyasatien

**Affiliations:** 1 Medical Science Program, Faculty of Medicine, Chulalongkorn University, Bangkok, Thailand; 2 Division of Infectious Diseases, Department of Internal Medicine, Faculty of Medicine, Prince of Songkla University, Songkhla, Thailand; 3 Department of Parasitology, Faculty of Medicine, Chulalongkorn University, Bangkok, Thailand; 4 Division of Dermatology, Department of Medicine, Faculty of Medicine, Chulalongkorn University, Bangkok, Thailand; 5 Medical Sciences, National Institutes of Health Ministry of Public Health, Nonthaburi, Thailand; 6 Excellence Center for Emerging Infectious Diseases, King Chulalongkorn Memorial Hospital, Thai Red Cross Society, Bangkok, Thailand; Institut Pasteur de Tunis, Tunisia

## Case Presentation

**Figure 1:**
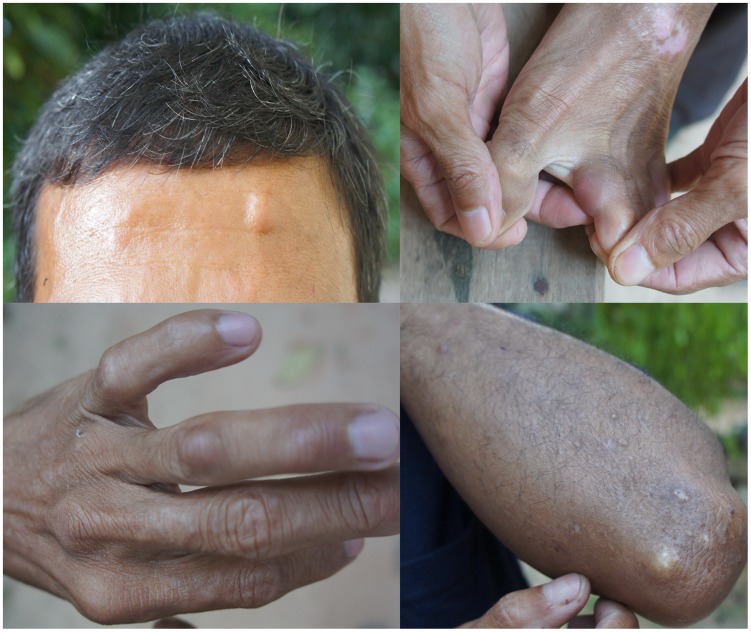
Nodules on brow, left second toe, left ring finger, and left elbow of nodular leishmaniasis case.

The patient was a 49-year-old male rubber planter living in southern Thailand who has had HIV infection for 10 years. He was diagnosed with disseminated cutaneous and visceral leishmaniasis 2 years previously and was treated with amphotericin B deoxycholate and itraconazole for leishmaniasis and with tenofovir, lamivudine, and nevirapine for HIV. Six months before this visit, he observed multiple nodules on his brow, left second toe, left ring finger, and left elbow (Fig. 1). His CD4+ Tcell count was 207 cells/mm^3^.

## Diagnosis and Treatment

**Figure 2:**
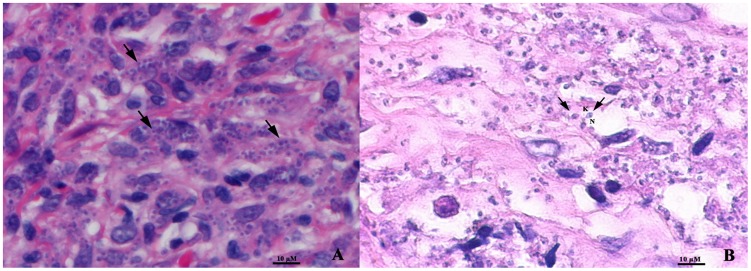
Numerous intracellular amastigotes (arrows) shown in a tissue biopsy of nodule from brow (A) and in a section of tissue submerged in Schneider's insect medium (B) with H&E staining. N, nucleus; K, kinetoplast (magnification x1000).

Diagnosis of relapsed *Leishmania siamensis* infection in this patient was performed by microscopic examination, culture, and polymerase chain reaction (PCR). Microscopic examination of tissue sections from his brow showed numerous intracellular organisms (Fig. 2A), and typical *Leishmania* amastigote parasites containing nucleus and kinetoplast were shown in a tissue biopsy that was submerged in Schneider's medium for two days and its sections then cut and stained with hematoxylin and eosin (H&E) (Fig. 2B). Numerous promastigotes were also observed in culture. PCR was performed using a primer set specific to the 18S rRNA gene of the internal transcribed spacer 1 (ITS1) of *Leishmania* spp. [1]. *L. siamensis* infection was identified by nucleotide sequencing and comparison with a sequence of *L. siamensis* reported previously (accession number JQ001751) and was shown to be 100% identical. After a final diagnosis of nodular leishmaniasis was established, the patient received 1 mg/kg/day of intravenous amphotericin B deoxylate for 28 days, followed by 300 mg of oral itraconazole twice a day for 5 months. The nodules regressed, and PCR detection of *L. siamensis* DNA in saliva and blood samples after the treatment was negative.

## Discussion

Leishmaniasis is a parasitic disease spread by the bite of infected female sand flies. The life cycle of *Leishmania* is initiated by sand flies feeding on the blood of an infected vertebrate host. Amastigotes of *Leishmania* transform into promastigotes in the digestive tract of female sand flies and are transmitted to a new vertebrate host during the next blood feed [2]. Currently, infection with the disease has resulted in approximately 0.2 to 0.4 million cases of visceral leishmaniasis and 0.7 to 1.2 million cases of cutaneous leishmaniasis [3]. Globally, leishmaniasis is a significant cause of morbidity and mortality in several countries. The disease is often a coinfection among HIV patients, tourists, refugees, and military personnel as well as among residents of endemic areas. Atypical clinical presentation of leishmaniasis has been described in AIDS patients, such as in a case of dermonodular and visceral leishmaniasis caused by *L. infantum* in an AIDS patient [4]. Leishmaniasis is usually found in HIV patients who have a CD-4+T cell level less than 200 cells/mm^3^ [5]–[9].

Autochthonous leishmaniasis cases in Thailand have dramatically increased in recent years [6]–[11]. The disease was reported in both immunocompetent and immunocompromised hosts, especially AIDS patients. Approximately 20 cases of autochthonous leishmaniasis have been documented, and most have been reported from southern Thailand [6]–[11]. Sukmee et al. [10] reported that autochthonous leishmaniasis in Thailand was caused by a novel species that later became known as *L. siamensis*. Phylogenetic analysis of three protein-coding DNA sequences showed a monophyletic clade that included both the *L. siamensis* and *L. enriettii* complex [7]. Surveys conducted in areas where *L. siamensis* infection has been reported in Thailand showed that *Sergentomyia (Neophlebotomus) gemmea* and *S. (Parrotomyia) barraudi* sand flies and black rats (*Rattus rattus*) were suspected to be a potential vectors and animal reservoirs for *L. siamensis*, respectively [12]. More recently, indigenous *L. siamensis* infection has been described in Myanmar in a patient who was treated with corticosteroids and in an AIDS patient [8], [13]. *L. siamensis* was also detected as a causative agent of cutaneous leishmaniasis in cows and horses in Germany [14], Switzerland [15], and the United States [16]. Patients infected with *L. siamensis* may present with classical clinical presentations of leishmaniasis, including visceral [9], [10], [11], diffuse cutaneous [8], [13], and overlapping diffuse cutaneous and visceral forms [6], [7], [8].

Diagnosis of *L. siamensis* infection relies on microscopy, culture, and PCR. Microscopic examination and culture are time consuming and require expertise to be reliable, while other screening tests for leishmaniasis such as enzyme-linked immunosorbent assay (ELISA), direct agglutination test (DAT), and rK39 dipsticks are not widely available. Diagnosis of *L. siamensis* infection is based on PCR and sequencing. Samples used for PCR for *L. siamensis* include blood, bone marrow, tissue, urine, and saliva. Recently, Phumee and colleagues demonstrated that saliva is a good source for PCR detection of *L. siamensis* DNA [8]. They also demonstrated that *L. siamensis* DNA levels in saliva decreased after treatment [8]. Saliva could thus be used as a biomarker to detect *L. siamensis* infection and follow treatment.

Several drugs are used for treatment of leishmaniasis, such as pentavalent antimonials, amphotericin B deoxycholate, lipid formulations of amphotericin B, miltefosine, and paromomycin. However, amphotericin B is the only drug available for treatment of *L. siamensis* infection in Thailand.

Recurrence of *L. siamensis* infection after amphotericin B therapy occurred in some patients. As documented in [9], a seronegative girl was successfully treated for a recurrent *L. siamensis* infection after amphotericin B treatment by extending amphotericin B therapy from 3 weeks to 5 weeks and following it with 6 months of prophylaxis.

**Boxes**

**Key Learning Points**

- Leishmaniasis caused by *L. siamensis* is an emerging disease in Thailand and Myanmar.
- *L. siamensis* infections were described in immunocompromised hosts such as individuals on systemic steroids and AIDS patients.
- Patients infected by *L. siamensis* can present with visceral, disseminated cutaneous, and overlapping of disseminated cutaneous and visceral forms.
- Diagnosis of *L. siamensis* can be performed by PCR using primers specific to the 18S rRNA gene of the ITS1, and saliva has been shown to be a good source of DNA for PCR.
- Amphotericin B is an effective therapy for *L. siamensis* infection. Although some patients have developed relapsed leishmaniasis after treatment, extended amphotericin B therapy followed by monthly prophylaxis for 6 months is effective.
